# Supplementary material for: Production of Sitobion avenae-resistant Triticum aestivum cvs using laccase as RNAi target and its systemic movement in wheat post dsRNA spray
Source: PLoS One. 2023 May 10;18(5):e0284888. doi: 10.1371/journal.pone.0284888 (PMC10171587; doi:10.1371/journal.pone.0284888)
Supplement: S1 Table — (DOCX) [file pone.0284888.s003.docx]

**Table S1. List of siRNA and dsRNA targets for Laccase gene by ERNAi (Boutros lab, E-RNAi-Version 3.2; Horn and Boutros 2010) (On Page 7 before Discussion).**

| **Reagent Quality** | | **siRNAs (19 nt)** | **On-target** | **Off-target** | **On-target** | **Efficient siRNAs** | **Sequence homology (e-value):** | **Average Efficiency Score** |
| --- | --- | --- | --- | --- | --- | --- | --- | --- |
| **Total siRNA targets** | | 144 | 114 | **0** | 30 | 143 | ACYPI006608(6e-81) | 57.95 |
| **S. no.** | **siRNA of Laccase1** | **Position in target** | **Length (BPs)** | **Intended target gene** | **Intended target transcript (hits)** | **On/off-target** | **siRNA location** | **Efficiency score** |
| 1 | GATGGAAAGAGTTGGAAAT | 345 | 19 | ACYP1006608 | ACYP1006608-RA (1) | 1/0 | EQ110779:52806..52824(+) | 97.75 |
|  | **Sequence homology (e-value) (**ACYPI006608(4e-04)&ACYPI002305 (0.024) | | | | | | | |
| 2 | CTTCCATCATTTCCTTTAT | 127 | 19 | ACYP1006608 | ACYPI006608-RA (1) | 1/0 | EQ110779:52509..52527(+) | 97.21 |
|  | **Sequence homology (e-value) (**ACYPI006608(4e-04)&ACYPI007038(0.093)&ACYPI008506(0.093) | | | | | | | |
| 3 | GGTCAATCGTTGAACTATT | 251 | 19 | ACYP1006608 | ACYP1006608-RA (1) | 1/0 | EQ110779:52633..52651(+) | 97.21 |
|  | **Sequence homology (e-value) (**ACYPI006608(4e-04) | | | | | | | |
| 4 | GGATGAGTTGTGATAATAT | 173 | 19 | ACYP1006608 | ACYP1006608-RA (1) | 1/0 | EQ110779:52555..52573(+) | 94.96 |
|  | **Sequence homology (e-value) (**ACYPI006608(4e-04)&ACYPI001739(0.093)&ACYPI008726(0.093) | | | | | | | |
| 5 | CCATCATTTCCTTTATTAT | 130 | 19 | ACYP1006608 | ACYP1006608-RA (1) | 1/0 | NA | 93.97 |
|  | **Sequence homology (e-value) (**ACYPI006608(4e-04)&ACYPI007054(0.093) | | | | | | | |
| 6 | GCTTCCATCATTTCCTTTA | 126 | 19 | ACYP1006608 | ACYP1006608-RA (1) | 1/0 | EQ110779:52508..52526(+) | 91.46 |
|  | **Sequence homology (e-value) (**ACYPI006608(4e-04)&ACYPI007038(0.093)&ACYPI008506(0.093) | | | | | | | |
| 7 | CAAATCATCCATTCCATTT | 299 | 19 | ACYP1006608 | ACYP1006608-RA (1) | 1/0 | EQ110779:52760..52778 (+) | 91.46 |
|  | **Sequence homology (e-value) (**ACYPI006608(4e-04) | | | | | | | |
| 8 | GTCAATCGTTGAACTATTT | 252 | 19 | ACYP1006608 | ACYP1006608-RA (1) | 1/0 | EQ110779:52634..52652(+) | 90.92 |
|  | **Sequence homology (e-value) (**ACYPI006608(4e-04) | | | | | | | |
| 9 | GATGGCGGATTTACAATAT | 460 | 19 | ACYP1006608 | ACYP1006608-RA (1) | 1/0 | EQ110779:52921..52939(+) | 90.92 |
|  | **Sequence homology (e-value) (**ACYPI006608(4e-04) | | | | | | | |
| 10 | CTTTCAAGCTTCCATCATT | 119 | 19 | ACYP1006608 | ACYP1006608-RA (1) | 1/0 | EQ110779:52501..52519(+) | 90.56 |
|  | **Sequence homology (e-value) (**ACYPI006608(4e-04) | | | | | | | |
| 11 | GATGAGTTGTGATAATATA | 174 | 19 | ACYP1006608 | ACYP1006608-RA (1) | 1/0 | EQ110779:52556..52574(+) | 89.93 |
|  | **Sequence homology (e-value) (**ACYPI006608(4e-04)&ACYPI001739(0.093)&ACYPI009122(0.093)&ACYPI008726(0.093) | | | | | | | |
| 12 | GTTATTTCATTGTCATATT | 510 | 19 | ACYP1006608 | ACYP1006608-RA (1) | 1/0 | EQ110779:53051..53069(+) | 89.93 |
|  | **Sequence homology (e-value) (**ACYPI006608(4e-04)&ACYPI004057(0.093)&ACYPI002228(0.093) | | | | | | | |
| 13 | GATTTACAATATTACGATT | 467 | 19 | ACYP1006608 | ACYP1006608-RA (1) | 1/0 | EQ110779:52928..52946(+) | 89.57 |
|  | **Sequence homology (e-value) (**ACYPI006608(4e-04)&ACYPI000672(0.006) | | | | | | | |
| 14 | GTGTACACACCACAAATTA | 91 | 19 | ACYP1006608 | ACYP1006608-RA (1) | 1/0 | EQ110779:52473..52491(+) | 88.67 |
|  | **Sequence homology (e-value) (**ACYPI006608(4e-04) | | | | | | | |
| 15 | GAAAGAGTTGGAAATCATA | 349 | 19 | ACYP1006608 | ACYP1006608-RA (1) | 1/0 | EQ110779:52810..52828(+) | 88.31 |
|  | **Sequence homology (e-value) (**ACYPI006608(4e-04) | | | | | | | |
| 1 | **DSRNA** | | | | | | | |
|  | **Intended Target gene:** | **Other targeted gene** | **Other targeted transcripts** | | **Intended target transcripts (hits):** | | **Sequence homology (e-value):** |  |
|  | ACYP1006608 | (NA) | (NA) | | ACYPI006608-RA (114) |  | (ACYPI006608(6e-81) |  |
|  | **Sequence Information (162 bps):** TGGATTCAAACAAGTGACGCATAAGTCTGAACAAGTGTACACACCACAAATTAACAAAATGTCTTTCAAGCTTCCATCATTTCCTTTATTATCTC  AAAGGAATATGATAGAACCTTGGATGAGTTGTGATAATATAAAGAAGGATTGCTCAAACGAATTCTG | | | | | | | |
|  | **F Primer+P** 5taatacgactcactatagggTGGATTCAAACAAGTGACGC 3  **R Primer+ P** 5taatacgactcactatagggCAGAATTCGTTTGAGCAATCC3 (**Tm[°C] :** 59.69) | | | | | | | |

Abbreviations. For Laccase: EQ110779: *Acyrthosiphon pisum* strain LSR1 SCAFFOLD7 genomic scaffold, whole genome shotgun sequence. ACYP1006608: gene and RA/- transcript. *A. pisum* laccase-1, gene and transcript variant X5, mRNA. % age homology score. *The lower the E-value, or the closer it is to zero, the more "significant" the match.
