# Supplementary material for: Production of Sitobion avenae-resistant Triticum aestivum cvs using laccase as RNAi target and its systemic movement in wheat post dsRNA spray
Source: PLoS One. 2023 May 10;18(5):e0284888. doi: 10.1371/journal.pone.0284888 (PMC10171587; doi:10.1371/journal.pone.0284888)
Supplement: S1 Raw images — (PDF) [file pone.0284888.s004.pdf]

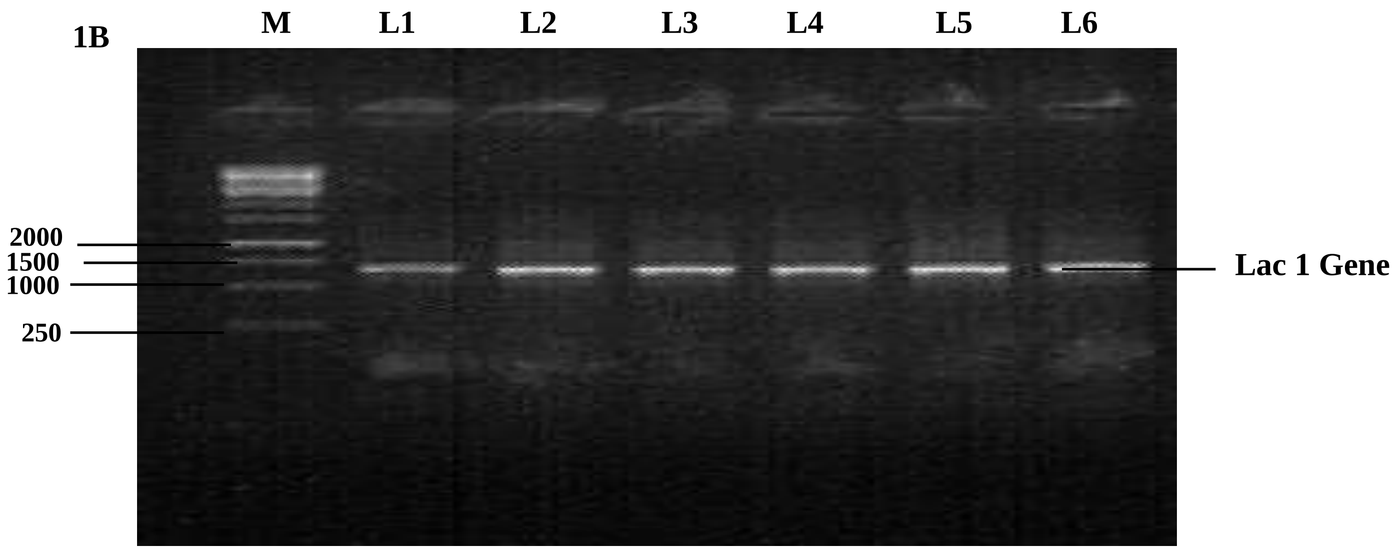

Fig 1. (1B) Reverse Transcriptase Polymerase chain reaction for (2A) Lac gene (615 bp) (L1) 1 Kb ladder, (L2-L6) Lac gene.

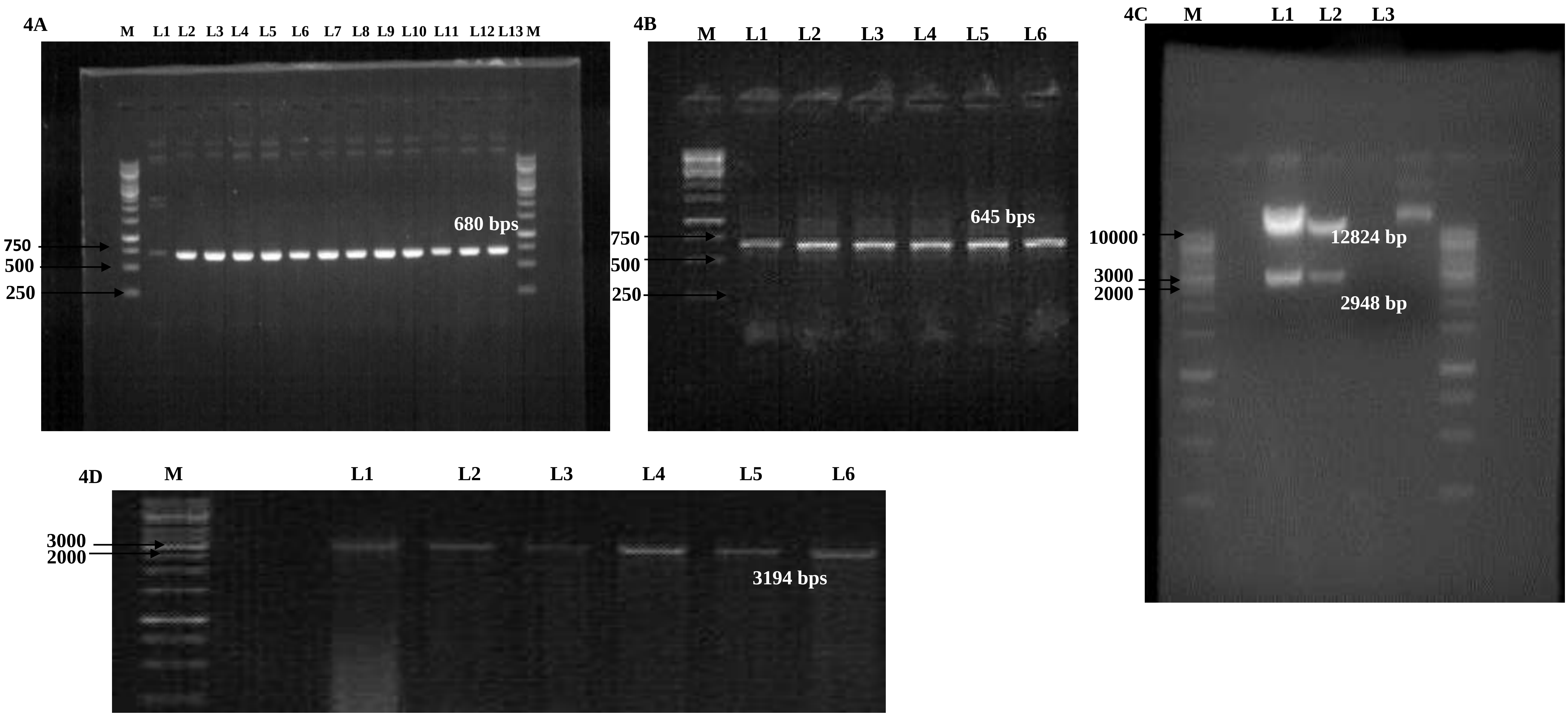

Fig 4. (**4A**) Colony PCR of RNAi-GG. M (1kb Ladder), L1-13 (PCR products of RNAi-GG). 4 A: RNAi-GG specific primers from pdk intron region (Tm=58; 680 bps) (**4B**) Lac1 confirmation M (1kb Ladder), L2-6. Lac1 with Vector-specific primers including adaptor sequences (Tm=53; 645 bps) (**4C**) RNAi-GG digestion with SacI, and SwaI (12824, and 2948 bps): M (1kb Ladder), L1-2 (Digestion products) (**4D**) PCR product using P-21 + P22 primers for whole insert confirmation (3194 bps), M (1kb Ladder), L1-6 (Whole insert) (Tm=55; 3194 bps).

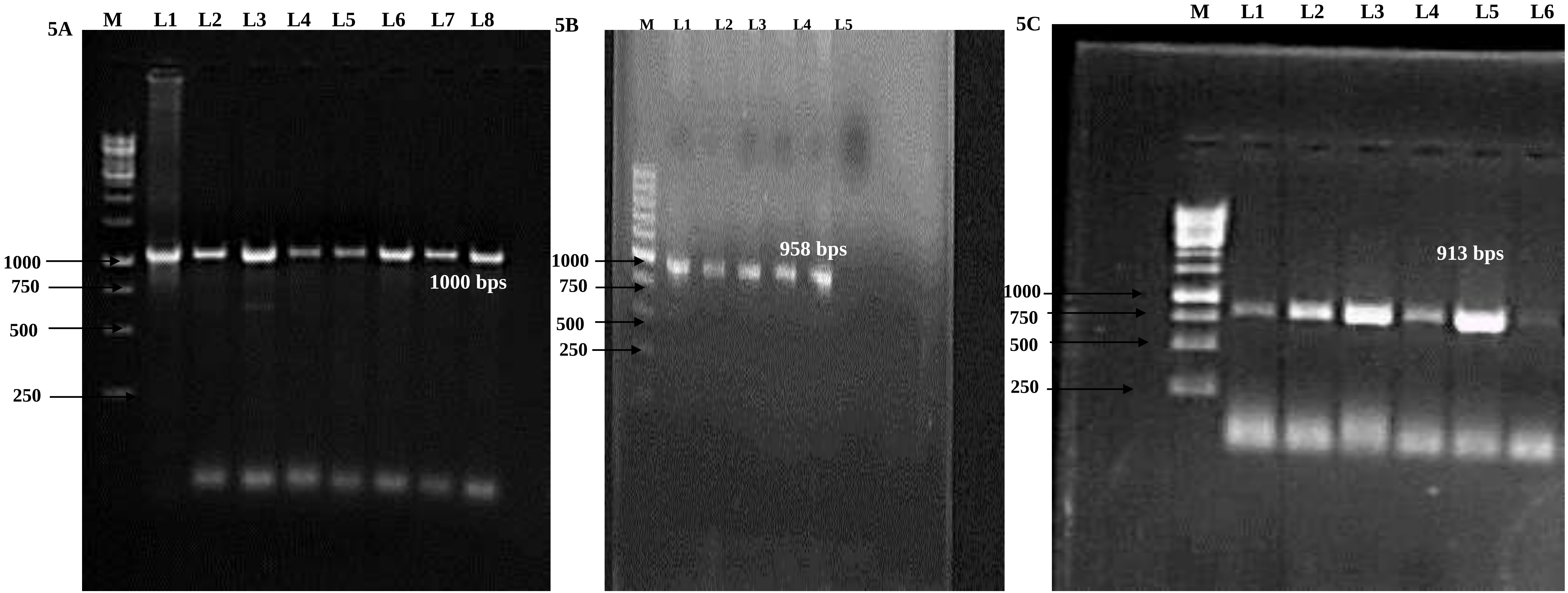

Fig 5. (5A) Lac 1 sense orientation confirmation by P-21 & 24 primers. M (1kb Ladder), L1-4 (Lac1 sense insert) (Tm=55; 1000 bps) **(5B)** Lac 1 antisense orientation confirmation by P-22 and P-25 primers. M (1kb Ladder), L1-5: Lac1 antisense insert (Tm=56; 958 bps) (**5C**) Lac1 antisense orientation confirmation by P-22 and Lac1 Reverse Primer. M (1kb Ladder), L1-2 (PCR antisense orientation insert) (Tm=56; 913 bps).

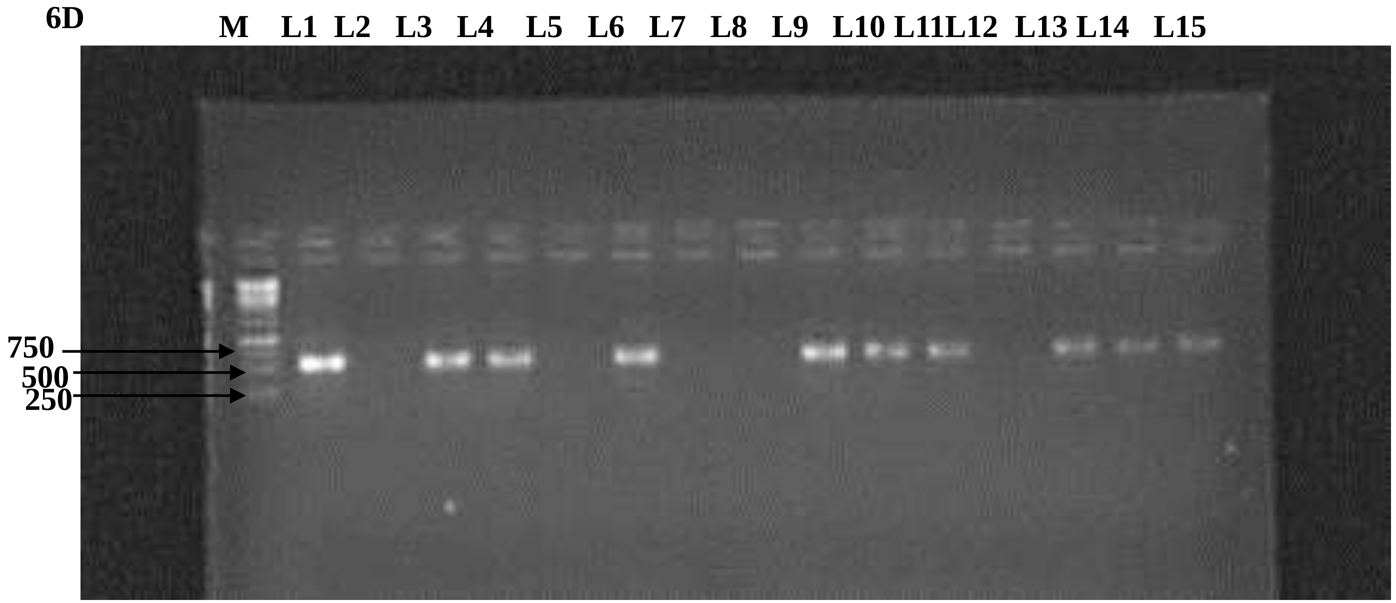

Fig 6. (**6D)** Lac1 confirmation in T1 *Triticum aestivum* cultivars. M (1kb Ladder), L1 (+ve control), L2 (-ve control), L3-4, 6 (Anaj 2017), L9-11 (Galaxy 2012), L13-15 (Punjab), L7, L8, & L12 (non-transgenic plants).
