# Supplementary material for: Production of Sitobion avenae-resistant Triticum aestivum cvs using laccase as RNAi target and its systemic movement in wheat post dsRNA spray
Source: PLoS One. 2023 May 10;18(5):e0284888. doi: 10.1371/journal.pone.0284888 (PMC10171587; doi:10.1371/journal.pone.0284888)
Supplement: Attachment — Submitted filename: PONE-D-23-00481.docx [file pone.0284888.s005.docx]

In this manuscript, authors evaluated efficacy of Lac. The manuscript is well written and meet the quality of this journal. I would recommend this manuscript for publication with minor revision.

Comments:

Why only authors selected dsRNA 20 ng μL−1 con. for bio-efficacy study?

1. The current title will not fit and need to be change

2. What is the efficiency of RT-PCR and qRT-PCR primers? are these validated? If yes, data should be included.

3. Line number 187-204 need to be merged and re written

4. Line number 150-157 and 234-242 are seems to be same.

5. All sub title are inappropriate and follow first letter as upper and the rest lower case

6. Fig.4, 5 and 6D, are looks bad quality and edited. submit original gel images.

7. Author tested six different concentrations of Acetosyringone and how is the transformation efficiency calculated and data should submit either in table or graphical
