# Supplementary material for: Production of Sitobion avenae-resistant Triticum aestivum cvs using laccase as RNAi target and its systemic movement in wheat post dsRNA spray
Source: PLoS One. 2023 May 10;18(5):e0284888. doi: 10.1371/journal.pone.0284888 (PMC10171587; doi:10.1371/journal.pone.0284888)
Supplement: Attachment — Submitted filename: Response to Reviewers.docx [file pone.0284888.s006.docx]

5 April 2023

PONE-D-23-00481

Golden Gate assembly for Sitobion avenae laccase silencing and its application as a biopesticide

PLOS ONE

Dear Editor,

Thank you for reviewing the manuscript Submitted to PLOS ONE. We are submitting a revised version of the manuscript addressing the points asked in the review process.

**Editorial Comments**

Please ensure that your manuscript meets PLOS ONE's style requirements, including those for file naming.

**Answer:** The manuscript is according to PLOS ONE requirement; Crosschecked.

2. In the Method section permits you obtained for the work. Please ensure you have included the full name of the authority that approved the field site access and, if no permits were required, a brief statement explaining why.

**Answer**: Work is performed in the Department of Biochemistry and Biotechnology, University of Gujrat, Gujrat Pakistan is added to the Methodology.

3. PLOS ONE now requires that authors provide the original uncropped and unadjusted images underlying all blot or gel results reported in a submission’s figures or Supporting Information files. This policy and the journal’s other requirements for blot/gel reporting and figure preparation are described in detail.

**Answer**: All original figures are attached (File Name: Original Figures).

4. In your cover letter, please note whether your blot/gel image data are in Supporting Information or posted at a public data repository, provide the repository URL if relevant, and provide specific details as to which raw blot/gel images, if any, are not available. Email us at [plosone@plos.org](mailto:plosone@plos.org) if you have any questions.

**Answer:** Not Applicable. All figures are in original figures.

5. We note that Figures 1 and 7 in your submission contain copyrighted images. All PLOS content is published under the Creative Commons Attribution License (CC BY 4.0), which means that the manuscript, images, and Supporting Information files will be freely available online, and any third party is permitted to access, download, copy, distribute, and use these materials in any way, even commercially, with proper attribution.

**Answer**: Figures 1 and 7 were the original figures that seems to be similar to some previous report. The figures are replaced with new ones.

6. Please include captions for your Supporting Information files at the end of your manuscript, and update any in-text citations to match accordingly.

**Answer**: Caption of supporting information is included & Highlighted.

**Answer to the Reviewers 1**

I am thankful to the reviewer for their valuable comments.

Comment 1. The manuscript must describe a technically sound piece of scientific research with data that supports the conclusions. Experiments must have been conducted rigorously, with appropriate controls, replication, and sample sizes. All data underlying the findings in their manuscript is fully available. The manuscript is presented in an intelligible fashion and written in standard English.

The conclusions must be drawn appropriately based on the data presented. Statistical analysis has been performed appropriately and rigorously.

**Answer:** Thanks for the comments. The conclusion had been changed.

**Reviewer #1**: The manuscript entitled "Golden Gate assembly for Sitobion avenae laccase silencing and its application as a biopesticide" reports a details investigation of RNAi strategy to control Sitobion avenae. The manuscript can be accepted in its present form, however, extensive proofreading is suggested to improve the readability of the manuscript.

**Answer:** Proofreading is done and checked by Dr. Shaukat Ali.

**Answer to the Reviewers 2**

Reviewer # 2: In this manuscript, the author evaluated the efficacy of Lac. The manuscript is well-written and meets the quality of this journal. I would recommend this manuscript for publication with minor revision.
Comments:
1. Why did only authors select dsRNA 20 ng μL−1 con. for the bio-efficacy study?

Answer: dsRNA 20 ngL-1 had been reported in previous reports for dsRNA assay. It is previously checked in OBP8 in our previous reports. It is cited now (Line # 155, 177).
2. The current title will not fit and need to be changed.

Answer: The Title is changed from “**Golden Gate assembly for *Sitobion avenae* laccase silencing and its application as a biopesticide” to**

**“Production of *Sitobion avenae-*resistant wheat using laccase as RNAi target and its systemic movement in *Triticum aestivum* post-dsRNA spray”**

**Short title:** changed from **“Laccase dsRNA for improved grain aphid resistance”** to **“Laccase silencing for improved grain aphid resistance”.**
2. What is the efficiency of RT-PCR and qRT-PCR primers? are these validated? If yes, data should be included.

Answer: Primer validation data is included now in the paper (Supp File 2).
3. Line numbers 187-204 need to be merged and re-written.

Answer: Line Numbers 202-217 are merged and rewritten.
4. Line numbers: 150-157, and 234-242 seem to be the same.

**Answer**: 160-172 are retained and Line number 232-242 are reduced to two lines (246-247).
5. All subtitles are inappropriate and follow the first letter as upper and the rest as lowercase.

Answer: Changed throughout & highlighted.
6. Fig.4, 5, and 6D, are looks bad quality and edited; submit original gel images.

Answer: Original Gel images are added.
7. Author tested six different concentrations of Acetosyringone and how the transformation efficiency calculated data should submit either in a table or graph.

Answer: The graph is included in the paper with acetosyringone affecting the transformation efficiency in Fig 6A. It is with the hygromycin selection the acetosyringone concentration is calculated. Transformation efficiency shown in the graph is based on selection.
